# 

**Published:** 1877-02

**Authors:** 


### COMMENCEMENT.

The thirty-first annual commencement of the Ohio College of Dental Surgery will be held in the college hall on Wednesday, March 7th, at half-past seven o'clock p. m.

The annual address will be delivered by Dr. E. J. Waye, of Sandusky, Ohio.

A cordial invitation is extended to all in any wise interested to be present.

### MISSISSIPPI VALLEY DENTAL ASSOCIATION.

The thirty-third annual meeting of the Mississippi Valley Dental Association will be held in the Ohio Dental College, at Cincinnati, commencing Wednesday, March 7th, 1877, at ten a. m. and continuing three days.

SUBJECTS FOR DISCUSSION.

I. Abrasion of the Teeth—Chemical and Mechanical.

II. Dental Caries: Cause and Prevention.

III. Mechanical Dentistry.

IV. Extraction of the Six-Year Molars: When Indicated?

V. Reflex Influences from Decayed Teeth: In what direction and to what extent do they occur?

VI. Treatment of the Teeth during Gestation and Lactation.

VII. Filling Teeth: Comparative value of different materials, and different methods of operating.

The Executive Committee would respectfully request a full attendance of the members.

A cordial invitation is extended to all in the profession to attend these meetings.

C. R. Taft,<br>F. A. Hunter,<br>H. A. Downing, } Executive Committee.

### MISSISSIPPI VALLEY MEETING.

The thirty-third annual meeting of the Mississippi Valley Dental Society will be held in the lecture room of the dental college in this city, beginning on Wednesday, March 7th prox., at ten o'clock a. m.

A programme and order of business has been prepared by the executive committee, which is published on another page of this number. The subjects presented for discussion are important, interesting and practical. It is desirable that every member should make some preparation on these subjects, and be able to contribute to the interest and profit of the meeting. Let all those who have new and valuable appliances bring them for exhibition.

Any new features or modes in practice should also be presented.

The indications are that it will be a large and interesting meeting. All members of the profession, whether members of the society or not, are cordially invited to be present; to all such the courtesy of the society is extended.

### COLLEGE ASSOCIATION.

The annual meeting of the Ohio Dental College Association will be held in the college on Tuesday, March 6th, 1877, at ten o'clock a. m. A full attendance of the members is very desirable, as important matters pertaining to the institution are to be considered and acted upon.

Let every member be present so far as possible.

A MONTHLY MAGAZINE DEVOTED TO THE INTERESTS OF

**THE DENTAL PROFESSION.**

*TERMS REDUCED TO $2.50 PER YEAR. SINGLE COPIES 25C.*

EDITED BY PROF. J. TAFT, D.D.S.

AND

**Published by SPENCER, CROCKER & CO., Ohio Dental Depot.**

The volume commences in January and closes in December.

The REGISTER being issued monthly is always fresh, therefore Indispensable to the practitioner who desires to keep posted up with the new inventions and improvements which are daily developed. Neither expense nor effort will be spared to give value and variety to its contents. Articles will be illustrated with well executed engravings whenever they will add to the interest or assist to explanation.

Its extensive circulation and frequency of issue make it one of the BEST DENTAL ADVERTISING MEDIUMS in the United States.

All subscriptions must begin with January or July.

Local or special notices, five lines or less, will be inserted for$3 each insertion; over five lines, 50 cts. per line.

All advertising bills payable in advance, or at furthest, after the issue of the first number containing the advertisement. All transient advertisements to be paid for in advance.

☞CONTRIBUTIONS SOLICITED.

Exclusively Editorial Communications should be addressed to Editor.

The latest number of the REGISTER may be ordered with or without other goods at any time, and all letters should be addressed to

***SPENCER, CROCKER & CO , Ohio Dental Depot, Cincinnati, O.***

